# Effect of housefly (*Musca domestica*) larvae on the growth performance and carcass characteristics of local chickens in Niger

**DOI:** 10.14202/vetworld.2022.1738-1748

**Published:** 2022-07-22

**Authors:** Bachir Hamani, Nassim Moula, Adamou Guisso Taffa, Idriss Hamidou Leyo, Chaibou Mahamadou, Johann Detilleux, Quynh Chau Dang Van

**Affiliations:** 1 Agronomy Faculty, Abdou Moumouni University of Niamey, BP 10 960 Niamey-Niger; 2 Fundamental and Applied Research in Animal and Heath, Departement of Veterinary Management of Animal Resources, Faculty of Veterinary Medicine, University of Liege, 4000 Liege, Belgium; 3 GIGA—Animal Facilities—ULiege—B 34, Liege, Belgium; 4 iCRA, Lawickse Allee 11, 6701 AN Wageningen, The Netherlands

**Keywords:** alternative feeds stuff, animals feeding, carcass, indigenous chicken, insect larvae, poultry diets, zootechny

## Abstract

**Background and Aim:**

The meat supply of local poultry for human consumption is greater than that of fast-growing poultry in Niger. However, meeting the protein needs of these local chickens is a major challenge due to the availability of protein sources and their cost. Nowadays, insect larvae such as houseflies are used and even recommended as animal feed; hence, the need to evaluate the effect of housefly (Musca domestica) larvae on the growth performance of local chickens. This study investigated the feeding effects of housefly larvae on the growth performance and carcass characteristics of local Nigerien chickens and determined the rate of fish meal substitution, in fresh or dry larvae form, whichever would be preferable.

**Materials and Methods:**

A total of 165 3-week-old local unsexed chickens of the salmon variety, weighing 120.3 ± 15.43 g, were used to evaluate the effect of housefly (M. domestica) larvae on their growth performance and carcass yield (CY). The experiment consisted of five treatments with three replicates, that is, 15 batches of 11 animals each. Five iso-protein-caloric diets were developed with 25%, and then 50% fish meal substitution with fresh and dried housefly larvae. The chicks were reared together during the first 3 weeks for their adaptation, during which they were fed an imported starter commercial feed, ad libitum. After that, they were weighed weekly for 12 weeks. Next, the body weights (BWs) were taken weekly for all chicks, feed daily intake and mortality were recorded daily, and average daily gain, feed conversion ratio (FCR), and viability rate were calculated. In the end, four chickens (two males and two females) per batch were slaughtered for the CY evaluation, breast meat, drumstick and tight (legs), and wings. Statistical analyses were performed using a linear mixed model for repeated data.

**Results:**

The weight, FCR, and carcass traits were unaffected by either the rate or larvae state. Conversely, the growth rate was improved, and feed consumption was increased. Notably, the chickens consumed more feed but grew faster with fresh larvae and at a higher substitution rate.

**Conclusion:**

This study reported that 50% fresh or dried housefly larvae substituted into the fish meal in growing local chicken’s diets had no effect on their BW, FCR, and carcass traits but increased the growth rate and feed consumption.

## Introduction

Poultry production is a source of food and income that contributes to food security and poverty alleviation in Africa [1, 2]. The use of fast-growing strains of chickens requires various input and technical knowledge unavailable to small-scale producers [3]. Breeding local chickens that are hardy and adapted to extreme conditions are widespread in African countries [3, 4]. These local chickens have fewer inputs and labor-intensive and are characterized by low production (growth and laying) [5, 6]. Although they are often impure breeds [7, 8], indigenous chickens have occupied a niche in an environment currently oriented toward a more sustainable production system dominated by agro-ecology and traditional or small-scale farming [9, 10]. Thus, traditional poultry accounts for more than 77% of the poultry population in many African countries [4]. Although modern poultry farming is emerging around cities in Niger, such as Niamey, the sector remains dominated by small-scale producers [10]. These indigenous chickens still provide major poultry products for consumption even in urban centers [6, 11].

In Niger, this sector area faces many constraints as in many southern countries, including poultry health problems [12], difficult climate conditions, lack of knowledge and skills among farmers, and the high cost of exclusively imported feed [3, 6, 13]. On average, feed costs are two-thirds of the costs associated with meat production of eggs and poultry [14]. Several efforts to substitute energy suppliers have been made, especially corn [15], and even imported protein sources such as soybean meal and fishmeal [16]. However, these protein sources are expensive and often scarce. Several studies have been conducted to find alternative protein suppliers to soybean and animal meals, such as fish or blood meal, which have health limitations [17]. Consequently, insects are a normal feed for poultry. Currently, interest in using insects for animal feed and human consumption has increased. Therefore, insect species or insect larvae have been experimented for animal feed, especially in monogastric animals [18]. This application is in the case of the darkling beetle larvae (*Tenebrio molitor*) [19, 20], black soldier fly larvae (*Hermetia illucens*) [21, 22], housefly larvae (*Musca domestica*) [23], locusts (*Ornithacris cavroisi*) [24], and many other species [18]. Notably, the insect species tested in West Africa are termites (*Trinervitermes* spp., *Odontotermes* spp., *Macrotermes* spp., and *Cubitermes* spp.) [23], locusts [24], and, recently, housefly larvae [22]. The housefly is a species found almost everywhere in the world. Its nutritional value for poultry is comparable to that of fishmeal [25], including a simple and low-cost rearing practice, given that it requires readily available substrates (household waste, cow dung, wheat bran, etc.) [26–28]. Housefly larvae have been substituted for soybean meal or fishmeal or incorporated into the feed for chickens [18, 29–37]. It has been shown that dried housefly larvae can substitute peanut meal in broiler diets without affecting their usual performances [29]. Notably, it can replace 60% soybean meal in broiler starter diets [30]. Housefly larvae can also substitute 33% of fishmeal without negatively impacting the broilers’ zootechnical performance [31]. Awoniyi *et al*. [32] found 25% as the efficiency rate of fish meal substitution with housefly larvae for average daily gain (ADG) and protein retention ratio. In a diet where the fish meal is 4% of the total diet, Okah and Onwujiariri [33] successfully replaced it with housefly larvae up to 50% of the fish meal amount in the diet of finisher broilers. A report has shown that 100% housefly larvae can replace 100% of raw materials of animal origin. Fresh housefly larvae induced the best performance in broilers in a cafeteria system with total substitution of fishmeal [35]. Adding 5% and 4% of housefly larvae to a standard diet for broilers in their starter and grower phases improved their performances significantly more than that in a standard diet alone [36]. Furthermore, Moula and Detilleux [18] reported that substituting insects for less than 10% of their total diet, excluding locusts, did not affect the growth performance of the birds. In a study in Ghana in 2002, Dankwa *et al*. [37] supplemented 30–50 g of fresh larvae per chicken in addition to the standard diet. They found significant improvements in the zootechnical performances of indigenous chickens compared with the performances obtained using a standard diet alone. However, these previous studies have focused on the rate of fresh and dried larvae in feeding broilers. Hence, it is interesting to address the feeding of chickens using a combination of fresh and dried housefly larvae with a substitution rate of fish meal in a native chicken strain.

Therefore, this study investigated the effects of feeding housefly larvae on the growth performances and carcass characteristics of local Nigerien chickens and determined the substitution rate of fish meal and the fresh or dry larvae that would be preferably incorporated.

## Materials and Methods

### Ethical approval

The experimental protocol was carried out as described in the following lines in compliance with the regulations applied in Niger, set out in the framework law on livestock breeding in the Republic of Niger [38].

### Study period and location

The study was conducted from June to September 2020. This study was conducted at the experimental farm of the Animal Production Department, Agronomy Faculty, Abdou Moumouni University, Niamey, Niger.

### Installations

Thirty boxes were set up in a building dedicated to experimenting. The boxes were arranged along the two main façades of the building, leaving a central corridor to ease access to each of them. Each of the 30 boxes was 3-m long and 1.6-m wide, with an area of 4.8 m^2^, which held about 38 broilers based on the broiler floor rearing recommendations for hot zones [39]. Rice husks were spread in boxes as litter. Each box was equipped with a drinker and a feeder, suspended by chains to facilitate adjustment according to the age of birds and to avoid water and feed wastage.

### Animals

The animals used were 165 3-weeks-old local unsexed chickens of the salmon variety bred from local salmon hens, which weighed 120.3 ± 15.43 g, and red roosters with black backgrounds purchased locally in villages within a 70-km radius of Niamey. The chicks for trial were reared together for the first 3 weeks, during which they were fed an imported starter commercial feed, *ad libitum* with 21.00% crude protein (CP), 2,840 kcal/kg dry matter metabolizable energy, 2.75% fat, 4.00% crude fiber, 1.00% calcium, and 0.45% available phosphorus (“Supreme Broiler Starter Mash,” Animal Care Services Konsult, Nigeria). The chicks weighed 120.3 ± 15.43 g at the end of the 3 weeks. This experiment comprised five treatments with three replicates, that is, 15 batches of 11 animals each. Consequently, there were five diets with three replicates, and the chicks were randomly allocated in batches of 11–15 non-adjacent boxes. Water and diets were provided *ad libitum* from the beginning to the end of the experiment. Notably, the identification of each chick was facilitated through individual monitoring using a colored ring.

According to the local poultry vaccine recommendations, all chicks were vaccinated against Newcastle and Gumboro diseases on days 7 and 10, respectively, with a booster on days 22 and 35.

### Diet formulation

Tables-1 and 2 show the five diets developed. Diet without larvae (DWL) is the control diet with no housefly larvae, and fishmeal is incorporated at 10% and 9.77% for the starter (3–6 weeks) and growers (7–14 weeks), respectively. The experimental diets comprised 25% dried larvae, 50% dried larvae, 25% fresh larvae, and 50% fresh larvae, in which dried and fresh larvae, respectively, substituted 25% and 50% of fishmeal. These diets were planned to be iso-energetic and iso-nitrogen for the same periods and meet the nutrient requirements of Leghorn-type chickens following the National Research Council recommendations [40]. The West African Poultry Feed Formulation Spreadsheet (TOAFA-Poultry) [41] was used to describe the diets, using the housefly larvae composition from the Feedipedia database [42]. The raw materials used were corn, wheat bran, peanut meal, fish meal, fresh and dried housefly larvae, two synthetic amino acids (lysine and methionine), bone meal, salt, and a mineral-vitamin supplement.

**Table 1:** Experimental diets composition for the starter period (from 3 to 6 weeks).

Composition (% Gross)	Unit	Treatments				

		DWL	25FL	25DL	50FL	50DL
Corn	%	63.47	61.50	61.50	59.54	60.00
Wheat bran	%	12.73	13.43	13.43	14.13	14.13
Fresh/dried larvae	%	0.00	2.50 (10.00)*	2.50	5.00 (20.00)*	5.00
Fish meal	%	10.00	7.50	7.50	5.00	5.00
Peanut meal	%	10.62	11.86	11.86	13.11	12.65
L-lysine	%	0.20	0.20	0.20	0.20	0.20
Dl-methionine	%	0.10	0.10	0.10	0.10	0.10
Bone meal	%	2.47	2.50	2.50	2.51	2.51
Salt	%	0.16	0.16	0.16	0.16	0.16
Mineral and vitamin premix	%	0.25	0.25	0.25	0.25	0.25
Calculated composition						
Metabolizable energy	Kcal/kg	2900	2900	2900	2900	2900
Crude protein	%MS	18.08	18.13	18.13	18.18	18.00
Fat matter	%MS	3.92	4.20	4.20	4.48	4.48
Crude fiber	%MS	3.47	3.58	3.58	3.70	3.70
Calcium	%MS	1.45	1.35	1.35	1.25	1.25
Phosphorus	%MS	0.69	0.66	0.66	0.63	0.63
Sodium	%MS	0.16	0.16	0.16	0.16	0.16
Chlorine	%MS	0.22	0.20	0.20	0.19	0.19
Lysine	%MS	0.88	0.86	0.86	0.83	0.83
Methionine	%MS	0.42	0.41	0.41	0.40	0.40

* In brackets, equivalent amount of fresh larvae, DWL=Diet without larvae, 25DL=25% dried larvae, 50DL=50% dried larvae, 25FL=25% fresh larvae, 50FL=50% fresh larvae

**Table 2:** Experimental diets composition for grower period (from 7 to 14 weeks).

Composition (% Gross)	Unit	Treatments				

		DWL	25FL	25DL	50DL	50FL
Corn	%	68.11	66.07	66.07	64.11	64.11
Wheat bran	%	12.07	13.39	13.39	14.26	14.26
Fresh/dried larvae	%	0.00	2.44 (9.76)*	2.44	4.89	4.88 (19.52)*
Fish meal	%	9.77	7.33	7.33	4.89	4.89
Peanut meal	%	5.00	6.05	6.05	7.21	7.21
L-lysine	%	0.10	0.10	0.10	0.10	0.10
Dl-methionine	%	0.20	0.20	0.20	0.20	0.20
Bone meal	%	4.00	3.68	3.68	3.60	3.60
Salt	%	0.50	0.49	0.49	0.49	0.49
Mineral and vitamin premix	%	0.25	0.25	0.25	0.25	0.25
Calculated composition						
Metabolizable energy	Kcal/kg	2890	2890	2890	2890	2890
Crude protein	%MS	15.55	15.60	15.60	15.66	15.66
Fat matter	%MS	3.89	4.18	4.18	4.46	4.46
Crude fiber	%MS	3.02	3.18	3.18	3.30	3.30
Calcium	%MS	2.02	1.79	1.79	1.66	1.66
Phosphorus	%MS	0.65	0.63	0.63	0.61	0.61
Sodium	%MS	0.29	0.28	0.28	0.28	0.28
Chlorine	%MS	0.43	0.40	0.40	0.38	0.38
Lysine	%MS	0.81	0.79	0.79	0.77	0.77
Methionine	%MS	0.40	0.39	0.39	0.38	0.38

* In brackets, equivalent amount of fresh larvae, DWL=Diet without larvae, 25DL=25% dried larvae, 50DL=50% dried larvae, 25FL=25% fresh larvae, 50FL=50% fresh larvae

Samples from each diet and protein raw material were taken before bromatological analysis according to AOAC International procedures [43] at the Feed and Animal Nutrition laboratory, Faculty of Agronomy, Abdou Moumouni University. Dry matter, CP, ether extract (EE), and ash were determined.

### Larvae production and drying

The study used housefly larvae produced in a room set up for this purpose by Hamidou Leyo *et al*. [26] at the Faculty of Agronomy, Abdou Moumouni University, Niamey. Five rearing cages (75 × 75 × 115 cm Bug-Dorm, Mega View Science, Taiwan) were used to maintain the breeding. Each cage had 25,000 housefly pupae reared inside, at a storage density of about 2.8 cm^3^ per fly, as described by Niu *et al*. [44]. Cotton soaked in sugar water used as adult food was placed in 83-mm diameter plastic containers. The cages were placed in a room close to the windows for maximum exposure to sunlight (12 h of light and 12 h of darkness). The room temperature was 27 ± 2.0°C, with a relative humidity of 60–70% [45]. Three to four days after adult emergence, plastic pots of 29 × 17 × 10 cm in size containing 50 g of fermented wheat bran were placed inside the rearing cages as an oviposition medium, as described by Holmes *et al*. [45]. Every 24 h, the oviposition medium was removed from the cages and placed in the larval development medium. The larval development medium, consisting of 1 kg of 70% moistened wheat bran, was placed in 50 × 35 × 15-cm trays on wooden shelves approximately 1.5-m high. After 5 d of larval development, the larvae were collected with a 3-mm sieve, packed in a storage bag, and stored in a freezer at −20°C. The rearing cages were cleaned at the end of each cycle (about 3 weeks), and adult fly colonies were renewed. A portion of the larvae was first thawed at room temperature, which generally varies between 23°C and 34°C from June- to September in Niamey, and then dried in an oven at 60°C for 24 h before use, which is a faster method to dry larvae than sun-drying [46]. The drying process reduced the larvae mass by 75%. Notably, the other part of the larvae, which is to be used in its fresh form, was stored in a refrigerator at 4°C to maintain its freshness. The raw materials used with these dried and fresh larvae were purchased in a local market in Niamey. The feed was prepared at an animal feed manufacturing unit (SAB-Niger, Niamey-Niger) with a grinder-mixer.

### Measures

Body weight (BW), feed daily intake (FDI), and mortality were recorded, whereas the average daily (ADG), gain feed conversion ratio (FCR), and viability rate (VR) were calculated. The BWs of all chicks were taken weekly using an electronic scale with 1-g precision. The ADG was measured in grams per week. The difference between the amount fed and the refusal collected daily was used to obtain the daily feed intake (DFI), expressed in gram (g). The quantities fed were adjusted daily to prevent refusals from exceeding 10% of the amount fed. However, they were reintroduced in the next day’s ration when refusals exceeded 10%. The FCR was calculated as the amount of feed consumed during a period to the weight gain during the same period. In addition, the VR was calculated as the number of birds alive at the end of a given period divided by the number of birds at the beginning of the same period. Furthermore, 60 chickens (four chickens per batch, two males and two females) were slaughtered for carcass characteristics evaluation at the end of the trial (at 15 weeks of age). The carcass characteristics evaluated were carcass yield (CY) as a percentage of BW and breast meat (BST), legs (tights and drumstick), and wings (W) as a percentage.

### Statistical analysis

Statistical analyses were performed using a linear mixed model for repeated data using the SAS method (Version 9.3, SAS Institute Inc., Cary, NC, USA). For BW and ADG, the model included the fixed effects of sex (male or female), age (from 1 to 12 weeks), diet (DWL, 25LS, 25LF, 50LS, and 50LF), and their two-way interactions, the fixed effect of the box nestled in the diets (n = 15), and the age × sex × diet interaction. Errors were assumed to be normally distributed with the Type 1-autoregressive structure. The same model was used for DFI and FCR without the effect of sex because they were measured at the box level. Statistical analysis was performed for all carcass characteristics using a linear mixed model with SAS mixed procedure (Version 9.3, SAS Institute Inc., Cary, NC, USA). Notably, the model included the fixed effects of sex and diet (DWL, 25LS, 25LF, 50LS, and 50LF) and their interactions. Errors were also assumed to be normally distributed with the variance component structure. Tests of difference between effect levels were declared significant at p ≤ 0.05.

## Results

No clinical signs of Newcastle disease or Gumboro disease were observed during the rearing period. Notably, this scheduled Newcastle disease and Gumboro disease prevention are suitable in the Niamey area where the monitoring was conducted.

### Diet compositions

Tables-3–5 show the bromatological analysis results of protein raw materials and experimental diets. The protein and ash content of the housefly larvae is comparable to those of the peanut meal. The fishmeal used in this experiment was more proteinaceous and contained more ash than the housefly larvae and the peanut meal used. In addition, the EE content of the larvae was similar to that of the fishmeal. However, the level of EE in the peanut meal found during this study was higher than that in housefly larvae and fish meal. A percentage average of 88.6 ± 5.05% in DM was found between diets for the starter period. In addition, the DM content was higher in the dried larvae diets (vs. fresh larvae) and the 25% larvae diets (vs. 50%). The CP, EE, and ash percentages were all similar between the diets. Additionally, the percentage average of dry matter was 88.7% ± 4.83 for the growth period. The DM content was higher in diets with dried larvae (vs. fresh larvae) and those with 25% (vs. 50%) of larvae. Furthermore, the percentages for CP (22.1% ± 0.58), EE (6.2% ± 0.66), and ash (9.3% ± 1.05), respectively, were similar between the diets.

**Table 3:** Analytical composition of protein raw material used.

Items	Units	Protein raw materials			

		Dried larvae	Fresh larvae	Fish meal	Peanut meal
DM	% Gross	96.70 ± 1.02	24.37 ± 0.20	90.69 ± 0.07	93.17 ± 0.09
CP	% DM	46.56 ± 0.16	-	56.14 ± 3.17	46.13 ± 1.12
EE	% DM	16.33 ± 0.02	-	18.95 ± 0.77	23.46 ± 0.61
Ash	% DM	5.88 ± 0.01	-	18.28 ± 1.76	5.45 ± 2.37

DM=Dry Matter, CP=Crude protein, EE=Ether extract

**Table 4:** Analytical composition of diets for the starter period (from 3 to 6 weeks).

Items	Units	Treatments					Means

		DWL	25FL	25DL	50FL	50DL	
DM	% Gross	91.1 ± 0.20	87.0 ± 0.53	92.1 ± 0.18	80.4 ± 0.03	92.2 ± 0.02	88.6 ± 5.05
CP	% DM	23.8 ± 0.59	23.3 ± 1.46	23.3 ± 0.38	24.0 ± 3.46	23.3 ± 2.55	23.5 ± 0.35
EE	% DM	9.0 ± 0.50	7.9 ± 0.35	8.9 ± 0.05	7.4 ± 0.26	7.5 ± 0.26	8.1 ± 0.75
Ash	% DM	8.3 ± 0.13	7.1 ± 1.30	7.1 ± 0.05	8.2 ± 1.28	6.4 ± 1.99	7.4 ± 0.79

DM=Dry matter, CP=Crude protein, EE=Ether extract, DWL=Diet without larvae, 25DL=25% dried larvae, 50DL=50% dried larvae, 25FL=25% fresh larvae, 50FL=50% fresh larvae

**Table 5:** Analytical composition of experimental diets for 7–14 weeks.

Items	Units	Treatments					Means

		DWL	25FL	25DL	50FL	50DL	
DM	% Gross	91.2 ± 0.07	87.0 ± 0.51	92.1 ± 0.15	81.0 ± 0.13	92.3 ± 0.15	91.2 ± 0.07
CP	% DM	22.5 ± 0.83	22.2 ± 0.28	21.1 ± 0.21	22.4 ± 5.11	22.5 ± 1.45	22.5 ± 0.83
EE	% DM	6.6 ± 0.11	6.7 ± 0.12	6.8 ± 0.65	5.2 ± 0.27	5.9 ± 1.38	6.6 ± 0.11
Ash	% DM	10.9 ± 0.79	9.2 ± 0.04	9.6 ± 2.35	8.7 ± 3.74	8.1 ± 0.48	10.9 ± 0.79

DM=Dry matter, CP=Crude protein, EE=Ether extract, DWL=Diet without larvae, 25DL=25% dried larvae, 50DL=50% dried larvae, 25FL=25% fresh larvae, 50FL=50% fresh larvae

### Overall effects of housefly larvae on the growth performance and carcass characteristics

Table-6 summarizes the effects of diet, sex, age, box, and their interactions on the different parameters measured. Mean ADG and FDI, adjusted for the effects in the model, were significantly different across diets and inversely to weight, FCR, and all carcass characteristics.

**Table 6:** Effects of diet (D), sex (S), age (A) and box (B) on zootechnical parameters (p-values).

Parameters	D	S	A	B	A × S	A × D	S × D	A × S × D
BW	0.3432	0.0001	0.0001	0.0109	0.0001	0.0001	0.6023	0.0089
ADG	0.0001	0.0001	0.0001	0.0001	0.0001	0.0001	0.0027	0.0511
FDI	0.0001	n.a.	0.0001	0.0001	n.a.	n.a.	n.a.	n.a.
FCR	0.1711	n.a.	0.0001	0.8037	n.a.	n.a.	n.a.	n.a.
CY	0.8880	0.5250	n.a.	n.a.	n.a.	n.a.	0.6090	n.a.
BST	0.6185	0.0003	n.a.	n.a.	n.a.	n.a.	0.1797	n.a.
Legs	0.1137	0.9051	n.a.	n.a.	n.a.	n.a.	0.2841	n.a.
W	0.3039	0.1534	n.a.	n.a.	n.a.	n.a.	0.8542	n.a.

BW=Body weight, A=Age, S=Sex, D=Diet, B=Box, ADG=Average daily gain, FDI=Feed daily intake, FCR=Feed conversion ratio, CY=Carcass yield, BST=Breast meat, Legs=Drumstick and tight, W=Wings, n.a.=Not available

### Effects of housefly larvae rate on the growth performance and carcass characteristics

**Figure-1:**
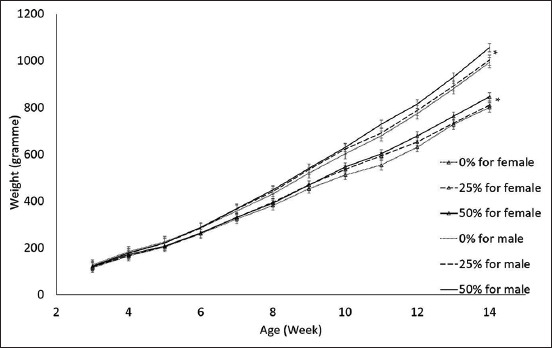
Body weight evolution by substitution rate and sex.

Table-7 shows the substitution rate results of fishmeal by the housefly larvae effect on BW, growth rate, feed consumption, and conversion ratio, CY, BST, legs, and W. Figure-1 shows the body evolution by the substitution rate and sex across the breeding period. The differences between the males and females were significant.

**Table 7:** Effect of housefly larvae substitution rate on zootechnical and carcass parameters.

Parameters	Units	Rates			p-value	
	
		0	25	50	25 vs. 0	50 vs. 0
BW	g	466.11 ± 11.53	475.66 ± 12.27	487.89 ± 11.51	0.5099	0.1250
ADG	g	10.06 ± 0.18	10.24 ± 0.19	10.77 ± 0.18	0.4095	0.0011
FDI	g	37.10 ± 0.38	36.55 ± 0.38	38.22 ± 0.38	0.2304	0.0157
FCR	-	3.43 ± 0.11	3.55 ± 0.11	3.28 ± 0.11	0.3792	0.2961
CY	%	66.69 ± 0.91	66.06 ± 0.91	66.58 ± 0.91	0.5710	0.9210
BST	%	18.98 ± 0.76	17.74 ± 0.76	17.68 ± 0.76	0.1888	0.1665
Legs	%	29.26 ± 0.97	31.40 ± 0.97	29.08 ± 0.97	0.0775	0.8816
W	%	13.17 ± 0.46	13.65 ± 0.46	12.94 ± 0.46	0.3972	0.6864

BW=Body weight, ADG=Average daily gain, FDI=Feed daily intake, FCR=Feed conversion ratio, CY=Carcass yield, BST=Breast meat, Legs=Drumstick and tight, W=Wings, g=Unit in gram, 25vs0=Comparison of the rate of 25% to the rate of the control, 50vs0=Comparison of the rate of 50% to the rate of the control

The overall ADG for the 25% rate was 10.24 ± 0.19 g/d, 10.77 ± 0.18 g/d for the 50% rate, and 10.06 ± 0.18 g/d for the control. Accordingly, a 25% rate improved the ADG by 0.18 g/d and a 50% rate improved the ADG by 0.71 g/d as compared to the control. This improvement was significant at a 50% rate (p = 0.0011) as compared to the control diet. Animals fed with a 25% substitution rate diet consumed an average of 36.55 ± 0.38 g/d feed, those fed with 50% consumed an average of 38.22 ± 0.38 g/d feed, and those fed the control diet consumed 37.10 ± 0.38 g/d feed. Notably, consumption was reduced by 0.55 g/d for the 25% rate compared with the control diet. However, the consumption was increased by 1.12 g/d for the 50% rate compared with the control (p = 0.0157). The overall FCRs for the 25% substitution rate were 3.55 ± 0.114, 3.28 ± 0.114 for the 50% substitution rate, and 3.43 ± 0.114 for the control. This result insignificantly differed between the experimental diets and the control diet. No differences between the carcass characteristics were also observed.

### Effects of housefly larvae forms on the growth performance and carcass characteristic

**Figure-2:**
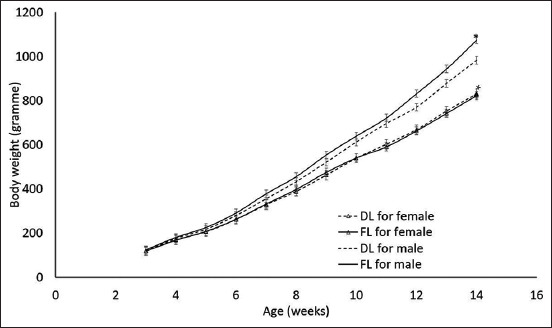
Weight evolution according to larvae form and by sex.

Table-8 shows the substitution rate of fishmeal by housefly larvae’s effect on BW, growth rate, feed consumption, FCR, CY, BST, legs, and W. Figure-2 shows BW evolution according to the larvae form and by sex across the breeding period. A significant difference between the growths of males and females was also recorded.

**Table 8:** Effect of housefly larvae form on zootechnical and carcass parameters.

Parameters	Units	Forms		p-value

		Dried larvae	Fresh larvae	
BW	g	489.50 ± 12.27	474.05 ± 11.51	0.1963
ADG	g	10.22 ± 0.18	10.80 ± 0.19	0.0017
FDI	g	36.64 ± 0.38	38.13 ± 0.38	0.0001
FCR	-	3.38 ± 0.11	3.45 ± 0.11	0.5776
CY	%	66.02 ± 0.91	66.63 ± 0.91	0.5060
BST	%	17.55 ± 0.76	17.88 ± 0.76	0.6793
Legs	%	30.55 ± 0.97	29.93 ± 0.97	0.5306
W	%	13.63 ± 0.46	12.96 ± 0.46	0.1516

BW=Body weight, ADG=Average daily gain, FDI=Feed daily intake, FCR=Feed conversion ratio, CY=Carcass yield, BST=Breast meat, Legs=Drumstick and tight, W=Wings, g=Unit in gram

The overall ADG for the animals that consumed dried larvae diets was 10.22 ± 0.18 g/d and 10.80 ± 0.19 g/d for those that consumed fresh larvae diet. Hence, fresh larvae significantly increased (p = 0.0017) the ADG by 0.58 g/d compared with dried larvae. Diets with dried larvae were consumed on an average of 36.64 ± 0.378 g/d, whereas those with fresh larvae were consumed on an average of 38.13 ± 0.38 g/d. Consequently, the consumption of fresh larval diets was increased significantly (p = 0.0001) by 1.49 g/d compared with the consumption of dried larvae diets. The overall FCR for diets with dried larvae was 3.38 ± 0.11 g/d, whereas that of diets with fresh larvae was 3.45 ± 0.11 g/d. Thus, the FCR for chickens fed with fresh larvae increased by only 0.07 points compared with the FCR for chickens fed with dried larvae. However, this variation was not significant (p = 0.5776). No significant differences in CY, BST, legs, and W were observed between chickens fed with dried larvae and fresh larvae.

At the end of this trial, viability was 100% for the control group, 99.50% for both rates, and even for the larvae form.

## Discussion

The recorded housefly CP of 46.56% ± 0.16 was in the range of the lower values reported by some authors [25, 29, 47–51], a higher value of 67.98% by Hwangbo *et al*. [50], and a lower value of 37.2% by Adeniji [29]. The recorded EE value of 20.84% ± 0.02 agrees with that found by Wang *et al*. (20.50%) [48]; however, it was greater than that reported by Pieterse and Pretorius (14.08%) [25] and lower than those by Adeniji (35.5%) [29], Odesanya *et al*. (31.76%) [49], and Hashizume *et al*. (29.56%) [47]. The ash value (5.88 ± 0.01) was similar to those reported by Adeniji [29], Hashizume *et al*. [47], and Wang *et al*. [48] but slightly lower than those reported by Pieterse and Pretorius [25], and Odesanya *et al*. [49]. Fish meal CP, EE, and ash were lower than those reported by Hashizume *et al*. [47] and Wang *et al*. [48], respectively. Therefore, this fish meal can be grouped with the low-protein fish meal of Heuze *et al*. [52]. In addition, the CP and ash in the peanut meal recorded in this study agree with those found by Batal *et al*. [53] and the value of the Feedipedia table [54]. However, we recorded a higher value of EE for peanut meal than these authors recorded. Comparing the chemical composition of housefly larvae, fish meal, and peanut meal showed that housefly larvae were intermediate between fish meal and peanut meal. In addition, more phenylalanine and tyrosine in housefly larvae were recorded than in the fish meal for the amino acid content [47]. In Pieterse and Pretorius’s study [25], threonine and valine were more abundant in the housefly larvae than in the fish meal. Lysine was more abundant in the fish meal than in the housefly larvae [49]. This author indicated a similar amount of methionine in these two raw materials. For the diet contents, the high moisture content in the fresh larvae caused the dry matter variation between fresh and dried larvae-based diets. Furthermore, this variation was due to the 5% versus 50% substitution rate of the fresh larvae diets. Notably, fresh larvae had high moisture content, as previously reported by Odesanya *et al*. [49]. In a study conducted in Burkina Faso, Sanou *et al*. [27] recorded a moisture content of 70.1–83.5% in the fresh housefly larvae. This study also discovered that oven drying at 60°C for 24 h reduced the larval weight by 75%. Slight variations in CP, EE, and ash showed a satisfactory balance between diets. Notably, the values recorded from the bromatology analysis were higher than those predicted in the formulation. These result differences were due to the nutrient composition variation of the raw materials. The Feedipedia tables showed a variation in the CP content of corn produced in sub-Saharan Africa from 2.0% to 12.4% on a dry matter basis, 23.8–62.9% for fishmeal, 38.5%–59.9% for groundnut meal, and 14.1–20.5% for wheat bran [52, 53–56]. These diets met the chicken feed requirement because the values determined by the bromatological analysis were higher than those expected from the formulation. This fact shows that the spreadsheet database of the West African poultry feed formulation used for this diet formulations underestimates one or more raw material compositions used for manufacturing experimental diets.

For growth performance, the local chicken in Niger responded well to sexual dimorphism, a known characteristic of several poultry species, including chickens [57]. Sexual dimorphism is manifested in growth parameters in chickens through better performance in males than in females [58]. The current study found that 50% substitution of housefly larvae in the fish meal of local chicken diets had no effects on BW. Therefore, fishmeal can be replaced in the feed of local chicken with 50% housefly larvae. This finding aligns with the reports of Ðorđević *et al*. [35], Okah and Onwujiariri [33], and Adeniji [29] for broilers. Importantly, Ðorđević *et al*. [35] verified a fish meal substitution of 50–100% by fresh and dried housefly larvae in four experimental diets. The control treatment was fed a standard diet, while the first and second experimental treatments were fed diets with 50% and 100% of fishmeal substituted with dried housefly larvae, respectively; the third treatment was fed a diet without fishmeal but supplemented with fresh larvae in separate feeders. The final BW comparison of chickens fed on 50–100% fish meal substituted diets with that of the chickens fed on a control diet showed no significant differences. Okah and Onwujiariri [33] substituted 20%, 30%, 40%, and 50% of fish meals with dried housefly larvae. He found that the BW of broilers in the control diet group was significantly lower than that in 20% and 30% of the diet groups but similar to the BW of broilers in 40% and 50% of substituted diet groups. Adeniyi [29] substituted groundnut meal of 25%, 50%, 75%, and 100% with dried housefly larvae meal in broiler diets and reported that no significant difference existed between the BW of the different treatments compared with that of the control. Moreover, Hwangbo *et al*. [50] found a linear increase in broilers’ BW with an inclusion rate of 10% and 15% of housefly larvae. This difference can be linked with the housefly amount substituted into the diets. In the Hwangbo *et al*. [50] experimental diets, there were 10% or 15% of housefly larvae; however, this study substituted 50% of the 10 and 9.77 g/kg fish meal, increasing the housefly larvae amount to 5% or 4.88% of the diets. The ADG of chicken was improved with a 50% substitution rate of fish meal by the housefly larvae, which could be due to the protein quality in the 50% substituted diet. The amino acids, which are more abundant in the housefly larvae than in fishmeal, were the limiting factor in the control diet. Importantly, threonine, one of the limiting amino acids for poultry, was found at relatively higher levels in housefly larvae than in the fishmeal [25]. This result agrees with a study by Téguia *et al*. [59] on broilers where the experimental groups were fed diets of 0%, 50%, and 100% of fishmeal substituted by dried houseflies larvae during the starter-growth phase. He recorded a linear increase in the daily weight gain with an increasing substitution rate. However, this result contradicts the report of Okah and Onwujiariri [33] in their studies on broilers, which recorded that the ADG and BW in chickens that consumed 40% and 50% housefly larvae substituted for the fish meal was similar to the ADG of the control group. Substituting fish meal with housefly larvae at 50% increased local chicken feed consumption. Notably, feed intake is usually linked to the growth rate [60]. The feed intake was also significantly increased as the ADG was improved considerably with a 50% substitution rate. The protein quality of these diets, especially threonine content [25], is the main reason for this increase. This trend was also observed by Téguia *et al*. [59] for a 50% partial substitution of fishmeal by housefly larvae during the finishing growth period of broilers. However, this contradicts the results of Okah and Onwujiariri [33], who reported a significant reduction in feed intake with an increased substitution rate.

Interestingly, some authors [26] propose that reduced feed intake is associated with the dark color of larvae, which changes the diet’s visual appearance, leading to its systematic refusal by birds. The FCR was used to measure diet efficiency. Remarkably, the substitution rate was unaffected by the FCR in this study. At these substitution rates, housefly larvae interact in the diet as fishmeal. Awoniyi *et al*. [32], Téguia *et al*. [49], Adeniji [29], and Ðorđević *et al*. [35] also observed this result. However, Okah and Onwujiariri [33] observed more efficient FCRs for diets containing housefly larvae than in the control diet. No effect of substitution rate was observed on any carcass characteristics. Housefly larvae did not affect CY, BST, legs, and W, which agrees with that of Elahi *et al*. [61] and Ren *et al*. [62]. Pieterse *et al*. [63], in another experiment, found an improvement in CY with 10% housefly larvae inclusion in the diet of broilers.

Furthermore, no effects were discovered in the BW of chickens that consumed diets with dried larvae compared with that of chickens that consumed fresh larvae diets. These results validate the results of Ðorđević *et al*. [35], who described the above, in addition to a fish meal-free diet, supplemented broilers with fresh housefly larvae in specific feeders—his third experimental group. These results are also aligned with those of Dankwa *et al*. [37], where the diet of local chickens was supplemented with 30–50 g of fresh housefly larvae, recording no difference in the BW between the supplemented and non-supplemented chickens. In addition, the fresh larvae improved ADG compared with the dried larvae. This improvement is possibly attributed to the destruction of some limiting amino acids in the fresh larvae diets during the drying of larvae into dried larvae diets, as heat can destroy some amino acids and reduce the digestibility of some raw materials through the Maillard reaction [64]. This trend deviates from the report of Ðorđević *et al*. [35]. The chickens’ variety and the monitoring time frame could explain the difference. Ðorđević *et al*. [35] worked with broilers for 6 weeks, whereas this study was conducted for 12 weeks on local chickens. Fresh larvae in local chicken diets induced a significant increase in feed consumption. This result reflects the evidence shown in Tables-4 and 5, wherein fresh larvae diets are more hydrated than dried larvae diets, especially as Scott [65] showed that broilers consume about 40% more of the hydrated than the dried diet. Chickens instinctively seek out scratching grounds to search for insects or their larvae. Therefore, insect or insect larvae are the natural feed for chicken [27]. This finding was also reported by Ðorđević *et al*. [35], who distributed fresh larvae in special feeders in addition to a diet without fishmeal to one of his experimental groups and substituted 100% fresh larvae with fish meal. He found that this group consumed more than the other groups that received a diet with dried larvae. Notably, several authors observed that the FCR was unaffected by the fresh and dried larvae forms [29, 32, 35, 59]. However, Dankwa *et al*. [37] observed more efficient FCRs for diets containing fresh housefly larvae than in control. This study showed no effect of larvae forms on all carcass characteristics.

Finally, for viability, neither substitution rate nor larvae form had any effect on the viability of local chickens, given that they all remained 100% viable during this study.

## Conclusion

The substitution rate of fish meal by housefly larvae up to 50% had no effect on the BW, FCR, and CY of local chicken, and in the housefly larvae forms, whether dried or fresh. However, the ADG and feed consumption increased by a 50% substitution rate of fresh larvae compared with that of the control. Consequently, it is possible to substitute fishmeal with fresh and dried housefly larvae in the indigenous poultry diet. Notably, fresh larvae are best suited to producers lacking drying or preservation facilities, especially given that fresh larvae involve no additional processing or preservation costs for a small-scale producer. However, the dried form has the advantage of integrating a complete diet for more efficient use by medium- to large-scale producers without the risk of screening the feed by animals.

## Author’s Contributions

CM, JD, QCDV, and NM: Conceptualization. BH: Methodology. NM, QCDV, and CM: Validation. JD: Formal analysis. BH, and AGT: Investigation. IHL: Resources. BH: Data curation. BH: Drafted the manuscript. QCDV and NM: Reviewed and edited the manuscript. CM: Supervision. JD: Project administration. All authors have read and approved the final manuscript.
